# Mathematical explanations for knee osteotomies: “Dear engineer, how would you explain it in a simple way?”

**DOI:** 10.1007/s00402-024-05366-7

**Published:** 2024-05-24

**Authors:** Marco Bechis, Federica Rosso, Marie Verocq, Bernardo Innocenti, Roberto Rossi, Davide Edoardo Bonasia

**Affiliations:** 1 Department of Orthopaedics and Traumatology, AO Ordine Mauriziano Hospital, University of Torino, Largo Turati 62, 10128 Turin, Italy; 2 Universite Libre de Bruxelles, Ecole Polytechnique de Bruxelles, Avenue F. Roosevelt 50 CP165/56, 1050 Brussels, Belgium

**Keywords:** Osteotomy, Mathematic explanation, Trigonometry, Tibial slope, Limb length discrepancy, Patellar height

## Abstract

**Introduction:**

Knee osteotomies are effective procedures to treat different deformities and to redistribute the load at the joint level, reducing the risk of wear and, consequently, the need for invasive procedures. Particularly, knee osteotomies are effective in treating early arthritis related to knee deformities in young and active patients with high functional demands, with excellent long-term results. Precise mathematical calculations are imperative during the preoperative phase to achieve tailored and accurate corrections for each patient and avoid complications, but sometimes those formulas are challenging to comprehend and apply.

**Methods:**

Four specific questions regarding controversial topics (planning methods, patellar height, tibial slope, and limb length variation) were formulated. An electronic search was performed on PubMed and Cochrane Library to find articles containing detailed mathematical or trigonometrical explanations. A team of orthopedic surgeons and an engineer summarized the available Literature and mathematical rules, with a final clear mathematical explanation given by the engineer. Wherever the explanation was not available in Literature, it was postulated by the same engineer.

**Results:**

After the exclusion process, five studies were analyzed. For three questions, no studies were found that provided mathematical analyses or explanations. Through independent calculations, it was demonstrated why Dugdale's method underestimates the correction angle compared to Miniaci's method, and it was shown that the variation in patellar height after osteotomy can be predicted using simple formulas. The five included studies examine postoperative variations in limb length and tibial slope, providing formulas applicable in preoperative planning. New formulas were independently computed, using the planned correction angle and preoperatively obtained measurements to predict the studied variations.

**Conclusions:**

There is a strict connection among surgery, planning, and mathematics formulas in knee osteotomies. The aim of this study was to analyze the current literature and provide mathematical and trigonometric explanations to important controversial topics in knee osteotomies. Simple and easy applicable formulas are provided to enhance the accuracy and outcomes of this surgical procedure.

## Introduction

Knee osteotomies are effective procedures to treat different deformities and torsional abnormalities to redistribute the load at the joint level, reducing the risk of wear and, consequently, the need for invasive procedures. Particularly, knee osteotomies are effective in treating early arthritis related to knee deformities in young and active patients with high functional demands [1, 2], demonstrating an average 5-years survival rate between 85 and 90% and a 10-years survival rate of 75% [3], with significant improvement in knee functional subjective and objective scores [4, 5] and a return to sport rate between 75 and 80% [6]. Knee osteotomies were initially performed to correct the weight-bearing axis on the coronal plane. However, despite the cut is made in the coronal plane, due to the complex biomechanics of the knee and the three-dimensional geometry of the tibia [7], significant changes may occur also in the other planes. It is not rare to inadvertently modify the tibial slope during a tibial osteotomy; if the slope is increased higher stress on the anterior cruciate ligament may be produced, increasing its risk of failure [8]. Furthermore, patellar height modification can be produced after a tibial osteotomy, with consequent anterior knee pain, a high risk of patellofemoral arthritis, and limitations in the range of motion [9, 10]. Lastly, increasing importance is being placed on preserving the obliquity of the joint line to obtain better outcomes and long survivorship in young patients, particularly after a High Tibial Osteotomy (HTO). Several studies highlight how excessive postoperative joint line obliquity can result in excessive shear forces [11], inferior outcomes [12], and lower 5-years surgical survival rate [13] after HTOs. Despite a recent systematic review [14] revealing a lack of standardized measurement methods in Literature with well-defined cut-offs and the real impact on postoperative outcomes, joint line obliquity undoubtedly remains a critical parameter in preoperative planning.

Precise and personalized preoperative planning is of paramount importance to achieve a biomechanically balanced knee in knee osteotomies, devoid of daily activity-related pain and discomfort during sports participation. The goal is to ensure satisfactory long-term outcomes to delay the need for partial or total prosthetic replacement as long as possible. Despite the excellent long-term results described in Literature [15, 16], there are still controversies, such as the best method for pre-operative planning, how to predict and control postoperative changes in tibial slope and patellar height and postoperative limb length discrepancies. All these aspects are strictly connected to different mathematical rules, and despite efforts made by authors such as Noyes et al. [7], Mihalko et al. [17] or Yamamuro et al. [18] to analyze and simplify the complex mathematics and trigonometry underlying these phenomena, the analyses and formulas provided may be difficult to understand for an orthopedic surgeon. The aim of this narrative review conducted by a team of orthopedic surgeons and an engineer, is to summarize the available Literature or give new simple explanations to the mathematical rules behind some of the major issues in knee osteotomies, to provide orthopedic surgeons with simple, reliable, and efficient tools to utilize for improving the preoperative planning and achieving better and more reproducible results.

## Materials and methods

An electronic search was performed on PubMed and Cochrane Library from January 1969 up to June 2023, to identify published original articles about mathematical explanations and osteotomy. “Knee osteotomy” was matched with the following terms: mathematical explanation, trigonometry, planning method, predictive formulas, limb length variance, tibial slope variance, patellar height change. English peer-reviewed articles which specifically addressed these topics with detailed mathematical or trigonometrical explanations were included. Three investigators (Marco Bechis, Marie Verocq and Federica Rosso) independently searched papers, screened titles, and abstracts of the retrieved articles reviewed the full-texts, and selected articles for their inclusion. Different specific questions were identified: (1) Is there a superiority of a planning method over the other and what is the underlying trigonometric explanation? (2) Is it mathematically feasible to predict the patellar height variation following the execution of a closing or opening wedge osteotomy? (3) How might an osteotomy impact the final length of the operated limb? (4) How can the tibial slope modification be predicted after a proximal tibial osteotomy?

The available Literature was summarized, when possible, with a clear mathematical explanation given by the engineer. Wherever the explanation was not already available in Literature, it was postulated by the same engineer.

## Results

**Fig. 1:**
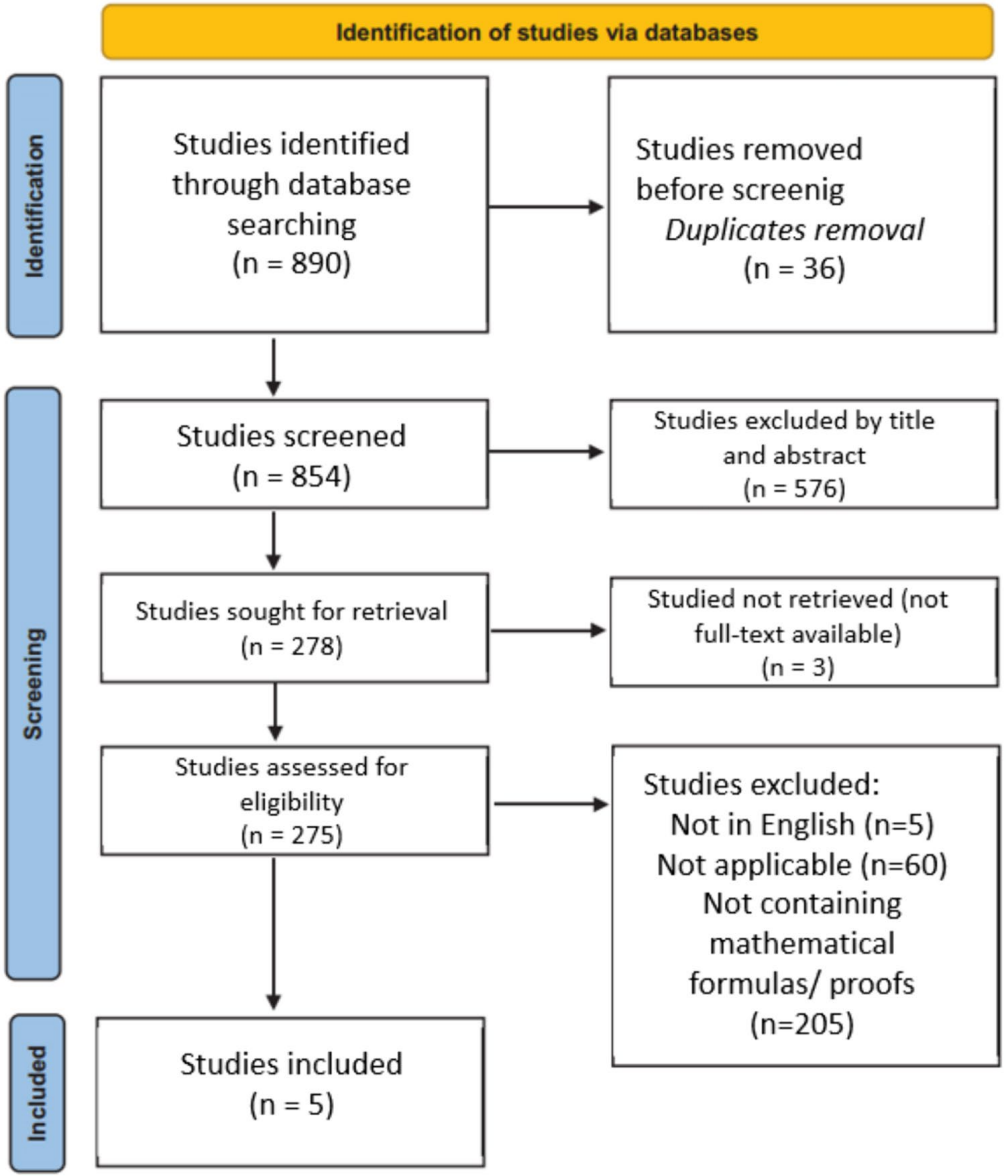
PRISMA guidelines

Eight hundred and ninety articles were returned during the initial search. There were thirty-six duplicate studies. The remaining 854 articles were analyzed, and of these, 576 were excluded based on title and abstract. The full texts of the remaining 278 were then reviewed, and based on inclusion criteria, 273 articles were excluded either for lacking mathematical formulas or proofs or for not specifically addressing the examined topics. Of the final 5 articles analyzed, two focused on post-operative variation in the length of the operated limb [17, 18], while the remaining three mathematically analyzed the variation in slope induced by osteotomy [7, 19, 20]. Figure 1 shows the inclusion process according to the Preferred Reporting Items for Systematic Reviews and Metanalyses (PRISMA) guidelines. Table 1 summarizes the included studies.

**Table 1:** Summary of the included studies

Authors/year	Aim	Subject	Findings
Noyes FR et al. (2005) [7]	CT images of the proximal tibia were digitized, and virtual tibial opening wedge osteotomies were performed The effects of the osteotomy on tibial slope and coronal correction were defined through algebraic calculation	35 patients	To maintain the preop slope, the anterior osteotomy gap should be one half of the posterior gap Every mm of gap error resulted in 2° slope change on average
Mihalko WK et al. (2001) [17]	Three different preoperative planning methods and three different types of tibial osteotomies (closing wedge, opening wedge, and dome type) were studied to numerically analyze the apparent leg-length change before and after osteotomy surgery	N/A (mathematical analysis)	Leg length variations of 0.5 to 3 mm were seen when the same osteotomy procedure was used with distinct preoperative planning approaches. Depending on the planning technique employed, variations in lower extremity alignment more than 7° might arise
Yamamuro Y et al. (2021) [18]	A retrospective case series simulation study was performed using a 3D preoperative planning software to simulate an opening wedge tibial osteotomy and examine the factors influencing leg lengthening	46 patients	True change in leg length showed a strong correlation with the opening width The mathematical formula provided showed a strong correlation with the measured leg length variation
Sariali E et al. (2009) [19]	A prospective non-randomized non-comparative study was performed to generate a mathematical model of the surgical procedure in order to plan the height of the medial opening wedge tibial osteotomy and to predict tibial slope changes	30 patients	The tibial slope can be kept unchanged after medial opening wedge osteotomy when using a carefully planned surgical technique The formula and the mathematical model were validated with perfect agreement between the model and the real values
Lee YS (2010) [20]	The study simulated HTOs using a three-dimensional polygon model of the leg to mathematically formulate medial and anteromedial opening gaps in the medial opening wedge HTO to achieve a targeted tibial posterior slope	30 patients	A formula was provided to estimate the opening gaps and opening angles to get a targeted posterior tibial slope with a medial opening angle osteotomy

The previously listed questions were individually addressed, and for each of them, simple mathematical formulas with their respective proofs and graphical representations were independently derived with the assistance of an engineer. In cases where formulas were already described in the literature, new calculations were performed independently to validate those formulas or derive new ones.

### Is there a superiority of a planning method over the other and what is the underlying trigonometric explanation?

The primary goal of knee osteotomies is to correct the weightbearing axis of the lower limb, thus achieving a Mikulicz line (a line connecting the center of the femoral head to the center of the ankle) passing through the center of the knee or slightly lateral to it, depending on the clinical and radiographic characteristics of the patient [21].

Two different planning methods are described in literature to calculate the desired correction: the Dugdale method [22] and the Miniaci one [23]. The Dugdale method is the most used. A line is drawn between the center of the femoral head and the desired correction point at the tibial plateau level, and a second line is drawn from the correction point to the center of the ankle. The angle between these two lines represents the desired correction to be achieved. The Miniaci method is slightly different. A line is drawn between the center of the femoral head and the desired correction point at the knee (new Mikulicz line), extending it to the ankle level. Subsequently, the hinge point is determined (e.g., in the case of an Opening Wedge High Tibial Osteotomy (OWHTO) located at the lateral cortical of the tibia, approximately 1–1.5 cm from the joint line), and two lines are drawn—one from the hinge point towards the center of the ankle and one from the same point towards the end of the new Mikulicz line. The angle between these two lines represents the desired correction.

Despite the simplicity and feasibility of the Dugdale method, several studies confirmed the superiority of the Miniaci method, which exhibits excellent intra/inter-observer correlation and higher accuracy. Siversten et al. [24] found that 14% of undercorrection could be attributed to the Dugdale method. Similarly, Ribeiro et al. [25] found that the corrective angle obtained with the computer navigation system and the Miniaci Method was 19% bigger compared to what was planned with the Dugdale method. However, both these studies are clinicals series with a retrospective evaluation of the reliability of the two methods, but without a mathematical explanation to this estimated undercorrection.

**Fig. 2:**
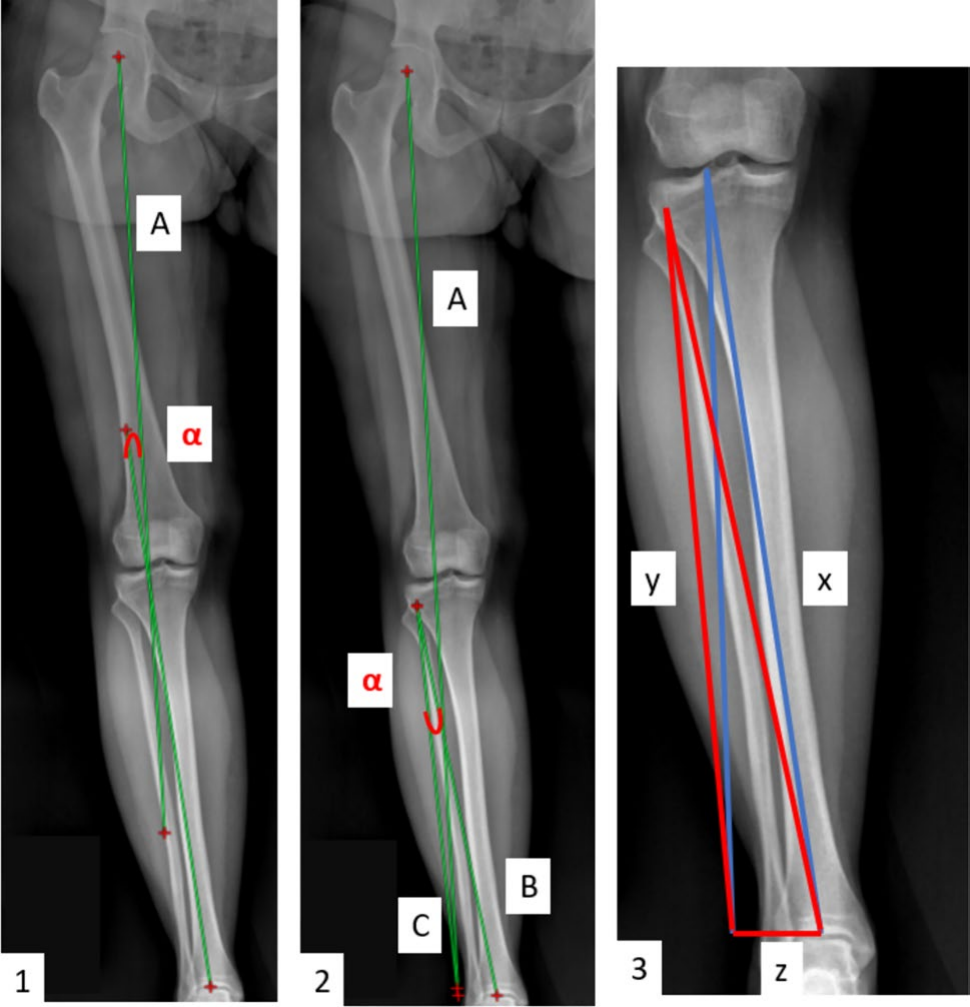
**1** Dugdale method: “A” is the line connecting the center of the femoral head and the target correction point on the tibial plateau (55% to 62.5% of the tibial plateau, corresponding to the lateral tibial spine); “B” is the line from the target point to the center of the ankle joint, the angle between them is the correction angle α. **2** Miniaci method: “A” is the line connecting the center of the femoral head and the target correction point on the tibial plateau, “B” is the line starting from the hinge point and ending over line A at the level of the ankle, “C” is the line from the hinge point to the center of the ankle joint, the angle between B and C is the correction angle α. **3** Graphic schematic representation: z is the amount of correction, y and x are the sides of the angle measured from the origin of the correction angle to the level of the ankle (in the image, red for Miniaci and blue for Dugdale)

The theoretical difference between the two methods is related to location of the correction angle at the tibial level. Dugdale calculates the angle with the apex located approximatively in the center of the knee at the level of the tibial plateau, while Miniaci places the correction angle at the hinge point (Fig. 2).

If the correction angle is located at the hinge point (Miniaci method), the amount of correction in mm (*z*) can be calculated with the following formula (law of cosines or cosine formula) [26]:

$$z^{2} = x^{2} + y^{2} - 2xy*\cos \alpha$$

Conversely, if the correction angle is located at the tibial plateau (Dugdale method) the same formula should be modified accordingly:

$$z^{\prime 2} = \left( {lx} \right)^{2} + \left( {ly} \right)^{2} - 2*lx*ly*\cos \alpha$$

where *z*′ is the amount of correction in mm and the factor *l* is the result of the following ratio*:* total tibial length/(total tibial length− distance between hinge point and the center of the knee (*d*)). The result *l* can be also expressed as a percentage (*l%)* dividing it by total tibial length.

$$l = \frac{total \,tibial \,length}{{\left( {total \,tibial \,length - d} \right)}} l\% = \frac{l}{total\, tibial \,length}$$

**Fig. 3:**
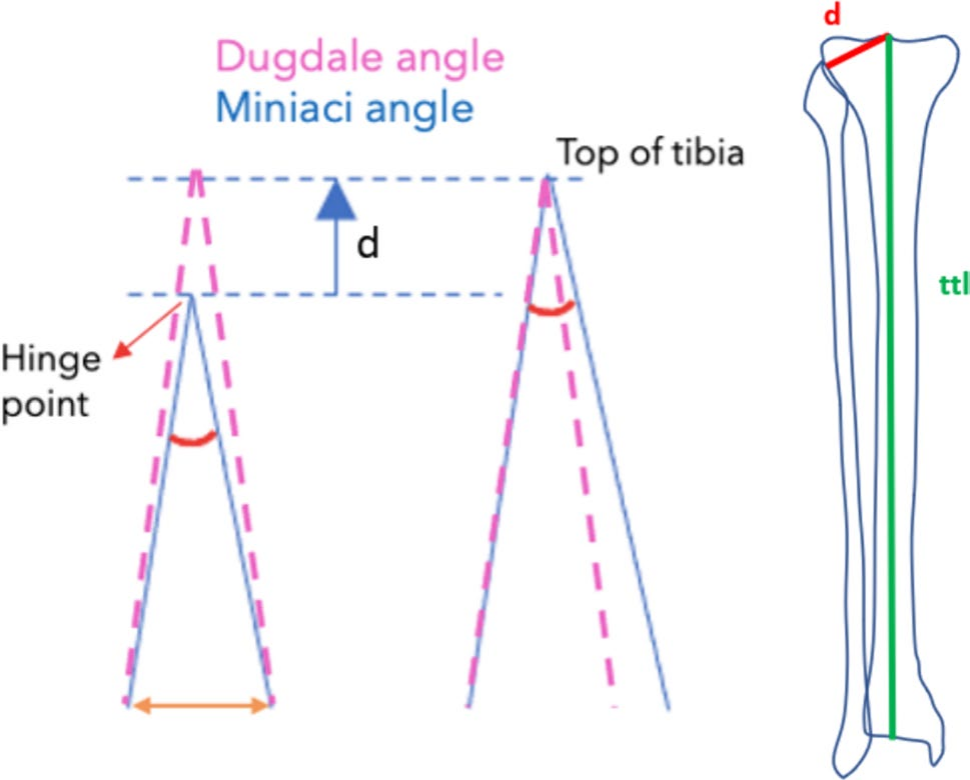
Schematic graphic representation of the difference between Miniaci and Dugdale planning methods. Pink interrupted lines represent the Dugdale method, while blue continuous lines represent Miniaci method. The letter *d* represents the distance between hinge point and the center of the knee. On the right side, *d* is graphically projected onto the tibia (red line), and the factor* l* is obtained from the formula *l* = *ttl/(ttl− d),* where *ttl* is the total tibial length (green line)

It is also possible to observe the difference from a simple graphical schematic representation (Fig. 3). On the left, the angle is drawn as in the Miniaci method, then the same angle is ideally moved near to the center of the as in the Dugdale method. Due to this translation, the length of the angle sides increases. By resolving the previous equation, it appears that by lengthening the angle sides of *l* %, to keep the same correction angle, the amount of correction should also increase by *l* %. Therefore, Dugdale tends to underestimate the correction angle of *l* %.

### Is it mathematically feasible to predict the patellar height variation following the execution of a closing or opening wedge osteotomy?

Knee osteotomies may produce undesired changes also on axial and sagittal planes [9]. Particularly, patellar height can be modified consequently to proximal tibial osteotomies. An excessively high patella can lead to instability and maltracking, conversely, a patella baja increases the risk of anterior knee pain and limitations in the range of motion [10].

**Fig. 4:**
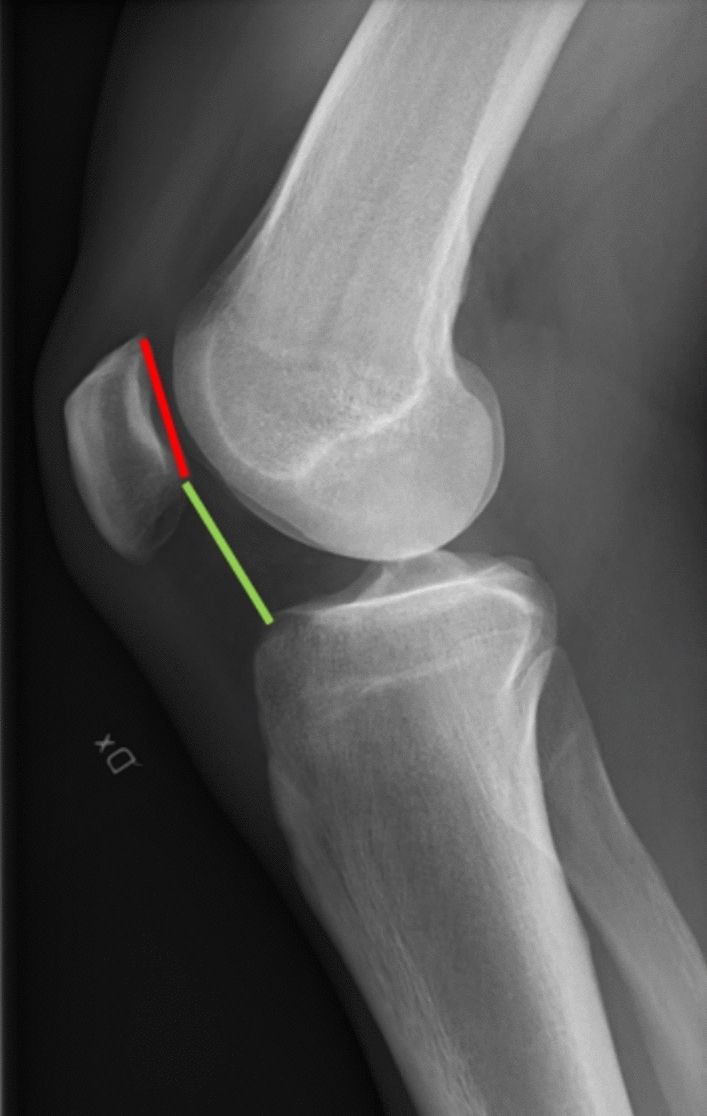
Caton-Deschamp index measured on a lateral X-ray. The distance between the anterior aspect of the tibial plateau and the most inferior aspect of the patellar articular surface (green line) is divide by the length of the cartilaginous articular surface of the patella (red line). Normal values are between 0.6 and 1.2

There are several methods for assessing patellar height based on a true lateral knee radiograph. However, various studies suggest that it is advisable to prefer indices that reference the joint line, such as the Caton-Deschamp index or the Blackburne-Peel index, over indices like the Insall-Salvati (ISI) which is measured by using the length of the patella and patellar tendon. Thus, ISI is unrelated to the change in patellar height determined by the joint line following HTO [27]. Particularly, the Caton-Deschamp index [28] is measured on a lateral x-ray with the knee ideally at 30° of flexion by dividing the distance between the anterior aspect of the tibial plateau and the most inferior aspect of the patellar articular surface by the length of the cartilaginous articular surface of the patella. Normal values are between 0.6 and 1.2 (Fig. 4).

It is well described a patellar height modification consequently to opening wedge (OWHTO) and closing wedge high tibial osteotomy (CWHTO), and these effects were more pronounced after OWHTO. CWHTO typically causes a shortening of the proximal tibia, which elevates the tibial tuberosity and raises the patellar height. Conversely, OWHTO causes the proximal tibia to move upwards, lowers the tibial tuberosity and reduces the patellar height. A recent meta-analysis by Lee et al. [29] compared the amount of patellar height variation in closing-wedge, monoplanar opening-wedge, and biplanar opening-wedge osteotomies with the the Caton-Deschamp index. The authors concluded about a decreased index by approximately 0.11 after monoplanar opening-wedge high tibial osteotomy (OWHTO), by 0.06 after ascending biplanar OWHTO, and by 0.01 after descending biplanar OWHTO. Conversely, the Caton-Deschamp index increased of about 0.02 after CWHTO.

Despite various techniques described for managing patellar height correctly [30], there is currently a lack of a mathematical explanation allowing to predict future changes in patellar height based on different osteotomy techniques.

**Fig. 5:**
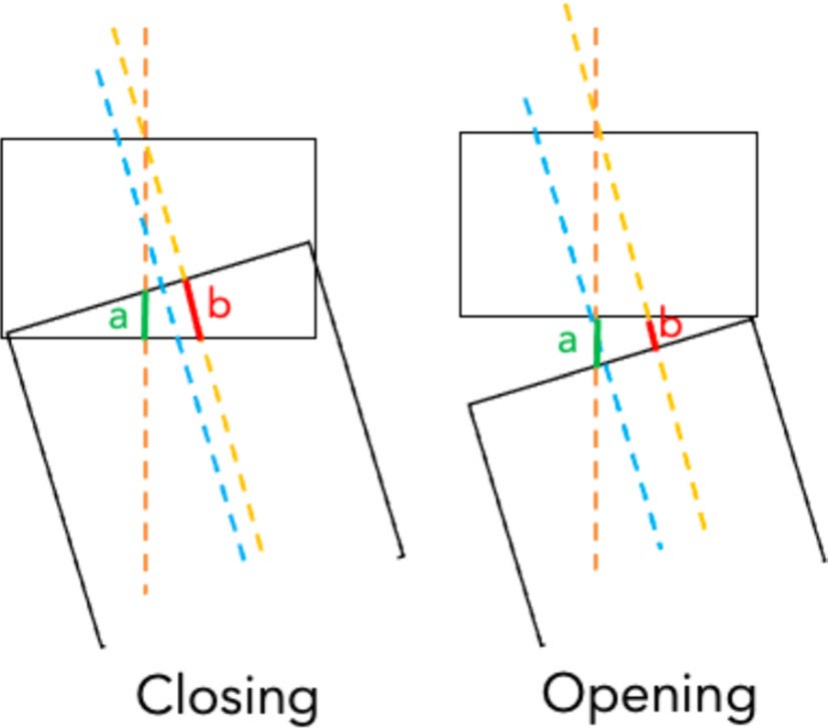
Schematic graphic representation. Orange line represents the native anatomical tibial axis, the blue line represents the new anatomical axis of the segment below the osteotomy cut and the yellow line is a transposition of the blue line but passing through the center of the knee and intersecting the orange line. Length *a* is measured on the orange line, length* b* is measured on the yellow line. The patellar height variation is expressed as *v* = *b− a.* For example, in the left figure* b* is higher than* a*, so *v* will result positive, meaning the patellar height will slightly increase, as in the case of a CWHTO. The opposite can be appreciated in case of an OWHTO (right figure)

To better understand the mathematics behind this phenomenon, a simple drawing simulation of what happens during opening and closing wedge osteotomy was performed. Two axes should be drawn on the tibia: the native anatomical axis and the new anatomical axis of the tibial segment below the osteotomy cut. A simulation of the desired correction was then performed by moving the lower segment around the hinge point, and two lengths were measured: length* a* (measured on the anatomical axis before rotation) and length *b* (measured on the new lower segment axis after rotation) (Fig. 5).

Therefore, it is possible to write the following equation, where *v* is the patellar height variation:

$$v=b-a$$

If *v* is negative, it means that patella height will decrease after the osteotomy. Conversely, if *v* is positive patellar height will increase after the osteotomy.

This mathematical explanation is in accordance with what can be found in different clinical studies, with OWHTOs resulting in an increased risk of patella baja, while CWHTOs by removing a wedge of bone above the tibial tuberosity result in an increased risk for patella alta. To reduce the risk of patella baja in OWHTO, biplanar descending osteotomies have been introduced, producing a constant position of the tibial tuberosity after surgery [29]. However, a mathematical explanation of this phenomenon, even if intuitive, is complicated due to the oversimplification of the tibial 3D anatomy a 2D and it may be better assessed with finite element studies or 3-dimensional reconstructions.

### How might an osteotomy impact the final length of the operated limb?

Changes in lower limb length can occur after knee osteotomies, due to both the corrective axial effect and the removal of a bone wedge in the case of a CWHTO, or, conversely, the creation of a gap in the case of an OWHTO [31, 32]. Several clinical and experimental studies have investigated this phenomenon, concluding that the degree of deformity, the correction angle, and the type of osteotomy are all factors influencing the postoperative length of the lower limb [17]. The meta-analysis conducted by Kim et al. [33] provided further detailed insight, with an average increased limb length of 7.6 mm after OWHTO. The lower limb lengthened in all patients in the OWHTO group, while in patients who underwent a CWHTO, both shortening and lengthening phenomena were observed, particularly in those with significant degrees of correction (> 10–15°). This suggests that, theoretically, the corrective effect on the mechanical axis of the lower limb is more influential than tibial thickness loss in cases of severe deformities.

These length variations can be clinically significant, as demonstrated also by Kim et al. who observed a postoperative increase in the length of the operated limb by more than 5 mm in approximately 90% of OWHTO. In contrast, this percentage dropped below 10% in the CWHTO group. As a results, a significantly greater proportion of patients in the OWHTO group were aware of the limb lengthening, compared with those in the CWHTO group [33]. This is in accordance with other studies in the literature that highlight how discrepancies exceeding 5 mm can result in alterations in gait biomechanics, chronic lower back pain, and limitations in daily activities [34, 35]. Consequently, it is advisable to consider the presence of any discrepancies in preoperative planning to select the most suitable osteotomy technique to minimize them.

Mihalko et al. [17] mathematically demonstrated that femoral and tibial length, and degree of the preoperative deformity may affect leg length after OWHTO. Yamamuro et al. [18] performed a three-dimensional analysis on more than 50 patients and determined that the leg length change can be predicted using the formula “change in total leg length = (opening width * 0.75) – 1.5”.

**Fig. 6:**
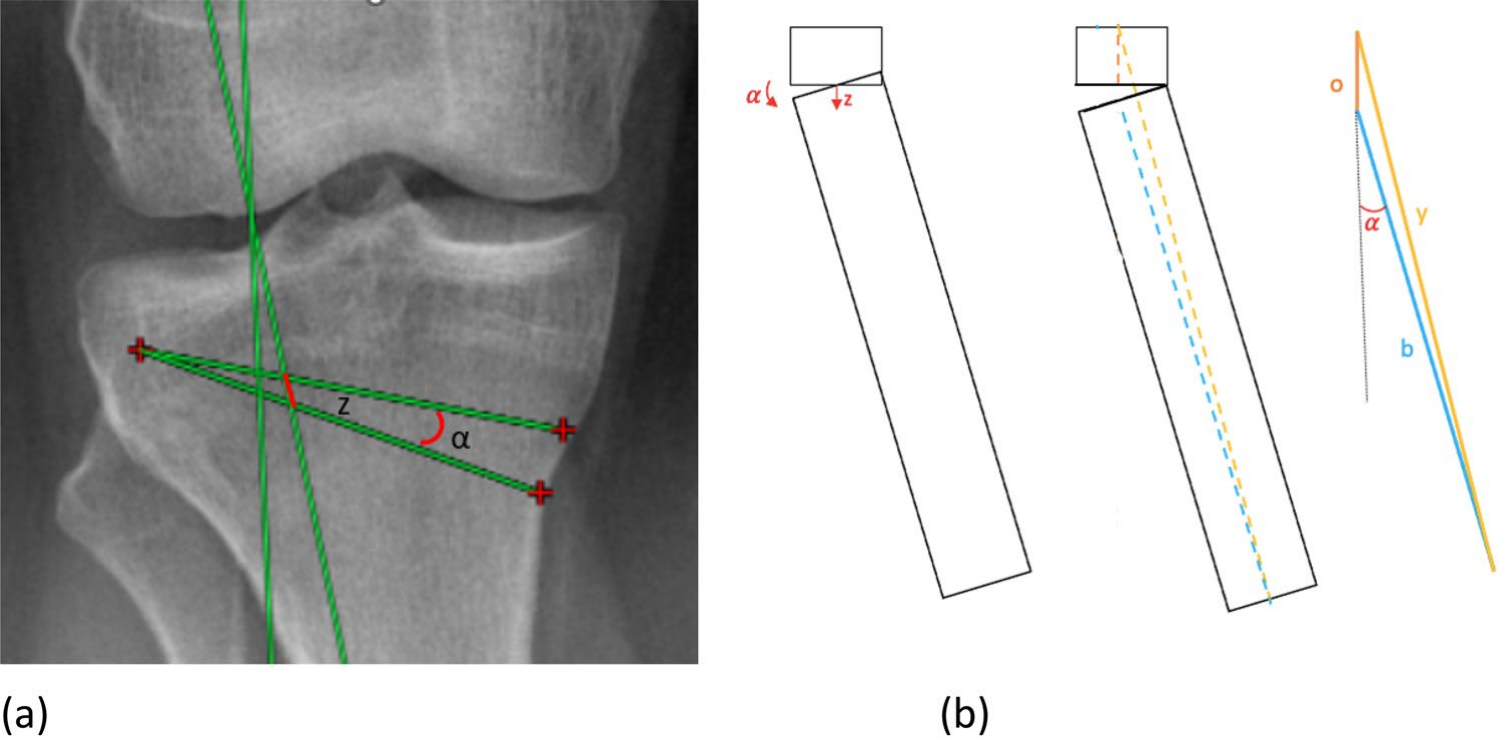
**a** α is the correction angle, *z* is the result of the projection of the correction angle over the new tibial mechanical axis. **b** Schematic graphic representation: α is the correction angle, *o* is the sum (or subtraction for closing) between the proximal part length and *z, b* is the length of the distal part

As the goal is to predict the limb length variation based on the correction angle, the first step is to find the amount of correction needed as previously described [22, 23]. Starting from the hinge point, this angle is then projected on the opposite tibial cortex with a direction equal to the osteotomy cut. The final step consists of measuring the length z, which can be described as the intersection between the correction angle and the new tibial mechanical axis (Fig. 6a). A verification of this length z is possible by measuring lengths x and y, the two sides of the angle, and using the following equation (law of cosines) [26]:

$${z}^{2}={x}^{2}+{y}^{2}-2xy*\mathit{cos}\alpha$$

$$z=\sqrt{{x}^{2}+{y}^{2}-2xy*\mathit{cos}\alpha }$$

Length *z* can be used to calculate the new tibial length after the osteotomy using the length of the tibial segment above the osteotomy and the length of the segment below it. It is then possible to reformulate the previous equation by replacing the values as follows:

$$y=\sqrt{{o}^{2}+{b}^{2}-2ob*\mathit{cos}\left(180-\alpha \right)}$$

where *y* is the total tibia length after surgery, *o* is the sum (or subtraction for closing) between the proximal part length and *z, b* is the length of the distal part, and $\alpha$ is the correction angle (Fig. 6b).

**Fig. 7:**
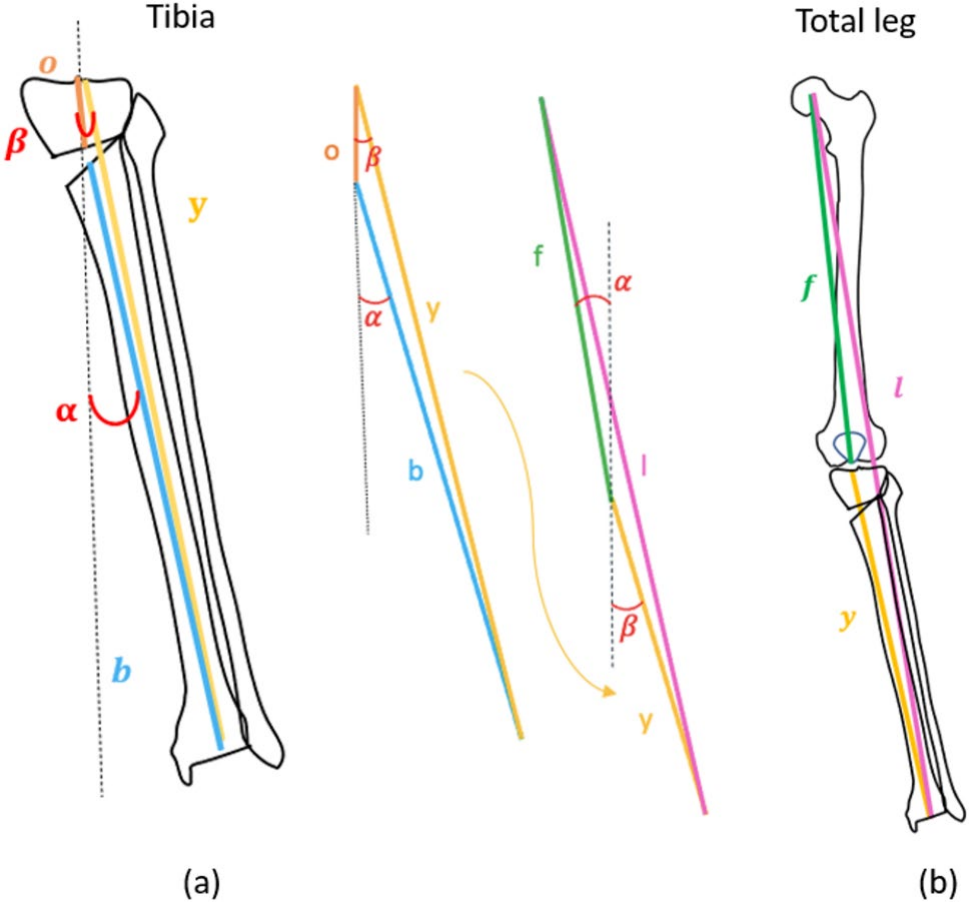
Schematic graphic representation of the previous formula with drawings and simulated projection on bone segment; **a** tibial side, **b** total leg. *l* is the whole limb length, *f* is the femoral length (from the center of the femoral head to the center of the knee), *y* is the new tibial length, α is the correction angle, and $\upbeta$ is the angle between the new mechanical tibial axis and the native tibial mechanical axis

These measurements can be used to predict the whole leg length. The previous equation can be further modified as (Fig. 7):

$$l=\sqrt{{f}^{2}+{y}^{2}-2fy*\mathit{cos}\left(180+\alpha \, -\beta \right)}$$

where* l* is the whole limb length, *f* is the femoral length (from the center of the femoral head to the center of the knee), *y* is the new tibial length calculated in the previous step, $\alpha$ is the correction angle, and $\beta$ is the angle between the new mechanical tibial axis and the axis of the tibial proximal part.

The $\beta$ angle can be calculated using the same data with the following formula:

$${b}^{2}={o}^{2}+{y}^{2}-2oy*\mathit{cos}\beta$$

$$\frac{{-b}^{2}+{o}^{2}+{y}^{2}}{2oy}=\mathit{cos}\beta$$

$$\beta =arccos\left(\frac{{-b}^{2}+{o}^{2}+{y}^{2}}{2oy}\right)$$

### How can the tibial slope modification be predicted after a proximal tibial osteotomy?

Posterior tibial slope (PTS) is defined as the angle between the vertical line of the tibial anatomical axis and the tibial plateau tangent, with average normal values between 5° and 10° [36]. A recent meta-analysis by Dean et al. [37]. highlighted how PTS can be measured using different imaging modalities (standard lateral radiographs, MRI, CT), locations (medial or lateral tibial plateau) and techniques (the vertical line can be tangent to the posterior tibial cortex, or can be a mid-diaphyseal line calculated with the midpoint technique or the circles technique). Therefore, during preoperative planning, it is crucial to acknowledge that each of these modalities has distinct normal range cut-offs.

PTS is a critical parameter in both knee replacement [38, 39], osteotomies [40, 41] and ligament reconstruction procedures [42, 43]. PTS significantly impacts different knee biomechanical parameters, including the range of motion, tension of the anterior and posterior cruciate ligaments (both native and reconstructed), flexion gap, and posterior femoral rollback [44]. Consequently, the tibial slope plays a crucial role in influencing the overall knee joint function and stability. For instance, as the PTS increases, the contact point between the tibia and the femoral condyle will move backward and the sagittal line of force will shift from the front to the back of the tibia. This will increase the tension on the ACL, increasing the risk of ACL injury and increasing the knee procurvatum [8]. On the other hand, if the PTS drops, the sagittal force line will advance and put more strain on the front of the tibial plateau. The posterior cruciate ligament (PCL) will experience more tension and the recurvatum will be increased, with a concomitant reduction of the flexion range [45].

As perfectly shown by Noyes et al. [7], the proximal tibial geometry can be approximated to a right triangle, where the oblique anteromedial surface of the tibia represents the hypotenuse that forms an angle of 45° with the posterior cortex, and the lateral cortex is perpendicular to the posterior plane of the tibia. Through mathematical formulas, this study has successfully determined that maintaining an anterior gap approximately half the size of the posterior one is crucial to avoid significant variation in the slope during an OWHTO. Furthermore, it was observed that every millimeter of gap change resulted in approximately 2 degrees of PTS variation. In the study by Song et al. the authors were able to preserve the original slope by keeping the anterior opening gap approximately 67% of the posterior one. In the case of CWHTO, attempting to remove a wedge of bone with approximately the same thickness both anteriorly and posteriorly and keeping the osteotomy strictly laterally and perpendicular to the anatomic axis would lead to a reduced risk of slope variation [46]. However, despite these recommendations, undesired slope variation is a relatively common phenomenon, as demonstrated by Nha et al. [41] meta-analysis of 27 studies. The authors concluded that PTS increases by approximately 2° degrees after OWHTO, whereas it decreases by about 2.35° after CWHTO. Conversely, there may be clinical situations in which the variation of the slope can be deliberately desired, such as to treat anterior [47] or posterior cruciate ligament chronic deficit in association to unicompartmental overload [48].

Being able to predict slope variation precisely during the preoperative planning may enhance the accuracy of the surgical procedure. Sariali et al. [19] utilized a three-dimensional mathematical modelling of a medial opening high tibial osteotomy with Cartesian references to predict the variation of the tibial slope after the procedure. To obtain the correction angle α they used the formula α = (180° − (hip-knee-ankle angle of the patient)) + β, where β is the future alignment of the patient (i.e., in case of a desired post-operative alignment of 3° of valgus the formula will result as α = (180°− HKA of the patient) + 3°). After obtaining the correction angle, the post-operative slope (P1) can be predicted by using the following formula (where P = is the pre-operative tibial slope):

$$\tan \left( {P1} \right) = \tan \left( {P0} \right)/\cos \alpha$$

**Fig. 8:**
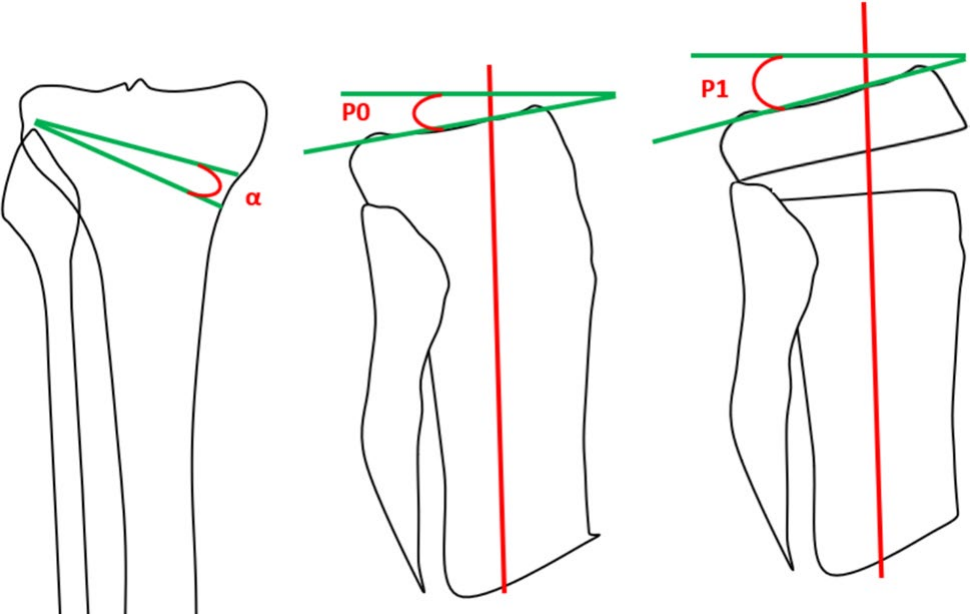
Schematic graphic representation α is the correction angle on the coronal plane, P0 is the pre-operative tibial slope and P1 is the post-operative tibial slope. The slope is calculated as the angle between the line perpendicular to vertical mid-diaphyseal line (red line) and the line tangent to the tibial plateau (green lines)

Lee et al. demonstrated in their study [20] that by simulating HTO with t a three-dimensional polygon model of the leg it is possible to generate a complex mathematical model able to predict a targeted post-operative tibial slope. Studies of this nature demonstrate how the intricate tibial geometry proves challenging to simplify, thereby rendering it more amenable to analysis through three-dimensional reconstructions or finite element studies. In attempting to investigate the mathematics behind the changes in tibial slope that occur subsequent to CWHTOs (which tend to reduce the slope) and OWHTOs (which tend to increase the slope) we used an oversimplified two-dimensional model and independently arrived at the same formula as described in the study by Sariali et al. [19]: *tan* (*P1*) = *tan* (*P0*)/*cosα* (Fig. 8).

## Discussions

Knee osteotomies are complex surgical procedures based on geometrical principles to achieve successful outcomes. As demonstrated in this narrative review, orthopedic surgeons can utilize mathematical measurements and modeling to make more accurate decisions about patient-specific corrections and achieve better long-term biomechanical effects. By analyzing the existing literature and applying trigonometric principles, simple and easy to use formulas applicable in clinical practice to enhance the accuracy of preoperative planning and consequently predict undesired outcomes have been formulated. Four different aspects have been investigated in this narrative review, including differences in planning methods, modification in patellar height, changes in tibial slope and limb length variations after proximal tibial osteotomies.

Consistent with prior studies [24, 25] albeit lacking a mathematical explanation, the Miniaci method demonstrated greater precision compared to the Dugdale method, which tends to underestimate the actual correction angle. A mathematical analysis of the difference between the two methods was conducted, concluding that the difference in between the methods is related to the different positioning of the origin of the correction angle, which differs by a distance ‘*d*.’ This distance can then be utilized to derive a factor ‘*l*.’ as described in the corresponding paragraph. When incorporated into the formula known as the cosine theorem [26], it allows to obtain the precise correction angle for the Dugdale method which, compared to the Miniaci method, would otherwise underestimate the correction by a value of ‘*l*’ (or expressed as a percentage, underestimates it by ‘*l%*’).

Both CWHTO and OWHTO can change patellar height. CWHTOs are found to result in an increase in patellar height, while OWHTOs may produce excessive patellar lowering [29], particularly in patients with preoperative patella baja, underlining the need for tailored surgical depending on deformity characteristics and possible consequences across all spatial planes. No mathematical explanations have been found in Literature on amount of patellar height modification after tibial osteotomies. Through a simple 2-dimensional graphical simulation of a CWHTO and an OWHTO, utilizing principles of trigonometry, a practical formula to predict whether the patellar height will be raised or reduced following the osteotomy was produced, helping surgeons to select the most suitable osteotomy type based on patient characteristics and deformity.

As indicated by various studies [7, 46], slope constitutes a parameter necessitating meticulous preoperative and intraoperative assessment due to its potential significant impact on knee biomechanics [47]. The complex three-dimensional geometry of the tibia proves exceedingly challenging to simplify into a two-dimensional graphical model. Both the studies of Sariali et al. [19] and Lee et al. [20] resorted to three-dimensional simulations and finite element studies to generate formulas capable of predicting the postoperative tibial slope variation. Particularly, the most readily applicable formula emerged to be the one described by Sariali et al. [19], and it was the same formula obtained in this study.

Ultimately, while osteotomies may focus more on the femoral or tibial aspects of the knee, it remains crucial to consider the alterations occurring at the hip, ankle, and comprehensively throughout the involved limb. Specifically, postoperative asymmetries can substantially affect patient quality of life [34, 35], and any preoperative length discrepancies must be considered when selecting the corrective technique [33]. Mihalko et al. [17] was the first to mathematically demonstrate how the amount of deformity and the degree of correction can significantly influence the postoperative length of the involved limb. Building upon this, Yamamuro et al. [18] employed a three-dimensional simulation to derive a formula capable of predicting the limb length change following an OWHTO. Leveraging the trigonometric principles, an equation applicable to both CWHTOs and OWHTOs was produced, enabling the prediction of limb length changes resulting from the osteotomy.

Finally, for greater simplicity, the initial questions were summarized into a conclusive table containing the derived formulas along with a brief explanation regarding their applicability (Table 2).

**Table 2:** Summary of the main mathematic formulas and brief explanations for each initial question

Topic	Question	Formula	Explanation
Planning method	Is there a superiority of a planning method over the other and what is the underlying trigonometric explanation?	Miniaci method: $${z}^{2}={x}^{2}+{y}^{2}-2xy*\text{cos}\alpha$$ Dugdale method: $$z^{\prime 2} = \left( {lx} \right)^{2} + \left( {ly} \right)^{2} - 2*lx*ly*\cos \alpha$$	Dugdale method tends to underestimate the correction angle by a factor *l,* where*: * $$l = \frac{total \,tibial\, length}{{\left( {total \,tibial \,length - d} \right)}}$$
Patellar height	Is it mathematically feasible to predict the patellar height variation following the execution of a closing or opening wedge osteotomy?	$v=b-a$	If *v* is positive, patellar height will increase (as in CWHTO) If *v* is negative, patellar height will decrease (as in OWHTO)
Post-op limb discrepancy	How might an osteotomy impact the final length of the operated limb?	New tibial length (y): $y=\sqrt{{o}^{2}+{b}^{2}-2ob*\mathit{cos}\left(180-\alpha \right)}$ New total limb length (l): $l=\sqrt{{f}^{2}+{y}^{2}-2fy*\mathit{cos}\left(180+\alpha \, -\beta \right)}$	OWHTOs tend to increase limb length more than CWHTOs, however the amount of correction is the most determining factor
Tibial slope	How can the tibial slope modification be predicted after a proximal tibial osteotomy?	$\tan \left( {P1} \right) = \tan \left( {P0} \right)/\cos \alpha$	Same formula as described by Sariali et al. [19]

For the legend of the terms used refer to the specific paragraphs

## Conclusions

This study highlights the critical role of mathematics in knee osteotomies. A good knowledge of the mathematical and geometrical principles behind this surgical procedure can help the surgeon in indicate for the most appropriate surgical technique, improving the clinical outcomes. Particularly, in young patients with early-stage osteoarthritis and high functional demands, a meticulously planned surgical technique for knee osteotomy, tailored to individual characteristics, may prove decisive or, at the very least, capable of significantly delaying the need for partial or total prosthetic replacement.

## Data Availability

All data supporting the findings of this study are available within the paper.
